# Draft Genome Sequence of a Chlorinated-Ethene Degrader, *Cupriavidus necator* Strain PHE3-6 (NBRC 110655)

**DOI:** 10.1128/genomeA.01743-15

**Published:** 2016-03-03

**Authors:** Kenta Yonezuka, Jun Shimodaira, Michiro Tabata, Shun Nagase, Daisuke Kasai, Akira Hosoyama, Atsushi Yamazoe, Nobuyuki Fujita, Masao Fukuda

**Affiliations:** a Department of Bioengineering, Nagaoka University of Technology, Kamitomioka, Nagaoka, Niigata, Japan; b Biological Resource Center, National Institute of Technology and Evaluation, Nishihara, Shibuya-ku, Tokyo, Japan

## Abstract

*Cupriavidus necator* strain PHE3-6 grows on phenol as a sole carbon source and cometabolizes *cis-* and *trans-*dichloroethenes and trichloroethene. Here, we report the draft genome sequence of PHE3-6, which provides insights into the degradation system of phenol and chlorinated ethenes.

## GENOME ANNOUNCEMENT

*Cupriavidus necator* strain PHE3-6 (NBRC 110655) was isolated from soil in Niigata, Japan. It utilized phenol as a sole carbon source and cometabolized chlorinated ethenes, such as trichloroethene (TCE), *cis*-dichloroethene, and *trans*-dichloroethene (tDCE). Chlorinated ethenes are known as environmental pollutants in soil and groundwater. TCE degradation by phenol degraders has been reported (1–6). However, tDCE degradation by phenol degraders has not been reported. Therefore, the degradation of chlorinated ethenes including tDCE in PHE3-6, is of importance for tDCE degradation is a novel activity among phenol degraders. To obtain insights into the degradation system of phenol and chlorinated ethenes in PHE3-6, the whole-genome shotgun sequence was performed.

The genomic DNA of PHE3-6 was sequenced by using paired-end sequencing with an Illumina MiSeq platform (Illumina, San Diego, CA, United States). The 4,892,926 reads obtained were assembled by Newbler version 2.6. The contigs were analyzed using the NCBI Prokaryotic Genome Annotation Pipeline (PGAP) (http://www.ncbi.nlm.nih.gov/genome/annotation_prok) to annotate protein-coding, rRNA, and tRNA genes. The draft genome of PHE3-6 has a total size of 7,267,813 bp with 66.33% G+C content and consists of 118 contigs ranging from 544 to 293,011 bp with an average coverage of 94× and an *N*_50_ length of 125,732 bp. The annotation revealed 6,722 protein-coding sequences and 59 RNA genes.

The 16S rRNA gene sequence of PHE3-6 has 98.56% and 100% identities with those of type strains *Cupriavidus taiwanensis* LMG 19424^T^ (CU633749) and *C. necator* N-1^T^ (CP002878), respectively. The analysis of average nucleotide identity (ANI) of PHE3-6 using the ANI calculator (http://enve-omics.ce.gatech.edu/ani) showed 88.64% and 99.49% ANI values with *C. taiwanensis* LMG 19424^T^ (CU633749 and CU633750) and *C. necator* N-1^T^ (AM260479 and AM260480), respectively. Thus, the phylogenetic affiliation of strain PHE3-6 was closely related to *C. necator* N-1^T^.

The results of genome sequencing indicated that PHE3-6 has a couple of gene clusters for phenol degradation, both of which contain orthologs of the multicomponent phenol hydroxylase subunit genes *dmpKLMNOP* in *Pseudomonas putida* CF600 (7). The *dmpKLMNOP* orthologs, which are located in the different contigs, showed amino acid sequence identities of 50.8% to 68.7% between their individual subunits and 40.7% to 65.6% with the corresponding subunits of CF600. The *dmpN* gene is known to encode the largest subunit of multicomponent phenol hydroxylase and contain catalytic domain. One of the *dmpN* orthologs showed an amino acid sequence identity of 99.6% with that of a phenol degrader, *C. necator* N-1^T^. The other *dmpN* ortholog showed an amino acid sequence identity of 87.8% with that of a TCE-degrading phenol degrader, *Burkholderia kururiensis* KP23^T^.

### Nucleotide sequence accession numbers.

The draft sequence of PHE3-6 has been deposited in the DDBJ/EMBL/GenBank databases under the accession number LMVF00000000. The version used here is the first version, LMVF01000000.
